# Genetic susceptibility of *IKZF1*, *ARID5B*, and *CEBPE* polymorphisms to childhood acute lymphoblastic leukemia in Chinese populations: a case-control study

**DOI:** 10.1016/j.gmg.2025.100084

**Published:** 2025-12-04

**Authors:** Xiaoqing Cao, Yong Wang, Yurou Kang, Muhammad Usman Abubakar, Yanli Yang, Daihua Fang, Bangshun He

**Affiliations:** a Department of Laboratory Medicine, Nanjing First Hospital, Nanjing Medical University, Nanjing, Jiangsu Province 210006, China; b Department of Laboratory Medicine, Nanjing Tongren Hospital, School of Medicine, Southeast University, Nanjing, Jiangsu Province 211102, China; c Department of Clinical Pharmacy, School of Basic Medicine and Clinical Pharmacy, Pharmaceutical University, Nanjing, Jiangsu Province 211198, China; d Department of Laboratory Medicine, Nanjing First Hospital, China Pharmaceutical University, Nanjing, Jiangsu Province 210006, China; e Department of Laboratory Medicine, Children’s Hospital of Nanjing Medical University, Nanjing, Jiangsu Province 210008, China; f Department of Blood Transfusion, The Affiliated Xuzhou Children’s Hospital of Xuzhou Medical University, Xuzhou, Jiangsu Province 221006, China

**Keywords:** Acute lymphoblastic leukemia, Single nucleotide polymorphism, SNP-SNP interaction

## Abstract

**Objective:**

This study aimed to evaluate the association of single nucleotide polymorphisms (SNPs) in *IKZF1*, *ARID5B*, and *CEBPE* with acute lymphoblastic leukemia (ALL) susceptibility in Chinese children.

**Methods:**

A case-control study was conducted involving 360 ALL patients and 398 healthy controls. Nine SNPs were genotyped, and their associations with ALL risk were analyzed using logistic regression under various genetic models. Multifactor dimensionality reduction (MDR) analysis was employed to investigate SNP-SNP interactions.

**Results:**

Seven of the nine SNPs were significantly associated with ALL susceptibility. Specifically, *IKZF1* SNPs (rs11980379, rs4132601, rs10272724) and a *CEBPE* SNP (rs4982731) were associated with an increased risk of ALL. In contrast, *ARID5B* SNPs (rs10994982, rs10821938) and another *CEBPE* SNP (rs2144827) were associated with a reduced risk. Stratified analyses revealed age- and sex-specific associations. MDR analysis identified significant SNP-SNP interactions, with a robust four-SNP model (rs10994982, rs2144827, rs10272724, rs10821938) showing the best predictive performance for ALL risk.

**Conclusion:**

Specific SNPs in *IKZF1*, *ARID5B*, *CEBPE* and their interactions are associated with childhood ALL susceptibility in Chinese populations, providing references for ALL risk stratification.

## Introduction

Acute lymphoblastic leukemia (ALL) is a malignant proliferation of lymphocytes blocked in the early stages of differentiation, which can invade the bone marrow, blood and extramedullary sites [1]. ALL have a high incidence among children, with a heterogeneous distribution by age, gender and geographic region [2], [3], [4], and the peak incidence of ALL is between 2 and 5 years of age, and more than 50 % are diagnosed before age 20 [5], [6]. Although environmental factors (radiation, chemical exposure, etc.) have been implicated in the development of ALL, genetic factors play an important role in susceptibility to ALL. Single-nucleotide polymorphisms (SNPs) are the most common form of genetic variation, accounting for more than 90 % of all known human genetic variations. A growing number of genome-wide association studies (GWAS) studies have identified SNPs associated with ALL susceptibility.

*IKZF1*, *ARID5B*, and *CEBPE* were the first group of genes identified by the GWAS performed by Treviño et al. and Papaemmanuil et al. [7], [8]. Since then, the association between SNPs in these three genes and ALL susceptibility has been extensively studied [9], [10], [11], [12], [13]. *IKZF1*, *ARID5B*, and *CEBPE* belong to different transcription factor families and play crucial roles in embryonic development, hematopoiesis and differentiation. *IKZF1* encodes the early lymphoid transcription factor IKAROS, a key regulator of lymphocyte development and homeostasis [14], [15]. Abnormalities in *IKZF1*, which may be deleterious to cellular homeostasis and mediate leukemic transformation, are common in ALL and are associated with a higher risk of relapse [16], [17], [18], [19]. *ARID5B* plays an important role in embryogenesis and growth regulation. It has been implicated in hematological system function and disease. Meanwhile, evidence from murine models demonstrates that *ARID5B* knockout disrupts normal B lymphocyte development [20]. Clinical study has observed *ARID5B* mRNA dysregulation is observed in malignancies such as acute myeloid leukemia (AML) [21] and acute promyelocytic leukemia [22]. Moreover, the pathogenic role of *ARID5B* in B-lymphoblastic leukemia was further illustrated by Zhao et al. [23] in a large-scale study. *CEBPE* is a critical regulator of terminal differentiation and functional maturation in myeloid cells, particularly neutrophils and macrophages [24]. Its dysfunction is implicated in both benign and malignant granulocytic disorders [25], [26], [27] and serves as an independent prognostic factor in AML [28]. Furthermore, numerous studies have established a strong association between *CEBPE* SNP and an elevated risk of ALL [24], [29], [30], [31].

Currently, studies focused on the SNPs *IKZF1*, *ARID5B*, and *CEBPE* and ALL susceptibility are primarily based on European, American, and Hispanic populations, with limited data from Asian cohorts and fewer SNPs loci examined. To assess whether these associations generalize across ethnicities, we conducted a case-control study in a Chinese population, analyzing nine previously identified SNPs across these genes from GWAS studies. Additionally, we investigated potential SNP-SNP interactions using multifactor dimensionality reduction (MDR) analysis.

## Materials and methods

### Study subjects

This case-control study recruited 360 ALL patients and 398 age- and sex-matched controls from Xuzhou Children’s Hospital and Nanjing Children’s Hospital. All patients were diagnosed with ALL based on MICM (Morphology, Immunology, Cytogenetics, Molecular biology), and controls were healthy individuals who participated in routine check-up. Information on patients and healthy controls was obtained from hospital medical records. The Ethics Committee of Xuzhou Children’s Hospital reviewed and approved the research protocol (Ethics No. 2023–05–09-H09).

### SNPs Selection

Candidate polymorphic sites within the *IKZF1*, *ARID5B*, and *CEBPE* genes were identified using the National Center for Biotechnology Information (NCBI) dbSNP database. The selection criteria for sites were a minor allele frequency (MAF) > 5 % and prior evidence suggesting a potential role in ALL susceptibility within certain populations. This study aims to expand research findings on these loci within the Chinese population by incorporating multiple genetic sites or further validating sites previously tested only in limited sample sizes. Finally, a set of nine SNPs were chosen as candidates for assessing their association with ALL risk in a Chinese pediatric cohort.

### DNA extraction and genotyping

Genomic DNA was isolated from anticoagulated whole blood samples using a Whole Blood Genomic DNA Isolation Kit (Xi’an GoldMag Biotechnology), following the manufacturer’s instructions. The purity and concentration of the extracted DNA were assessed on a Nanodrop 2000 spectrophotometer (Thermo Fisher Scientific, USA). Samples meeting the quality thresholds, a minimum concentration of 20 ng/μL and an A260/A280 ratio between 1.7 and 2.0, were selected for subsequent genotyping. Prior to genotyping, qualified DNA samples were normalized to a uniform concentration. Genotyping was performed using the Sequenom MassARRAY platform (Agena Bioscience). The experimental procedure involved PCR amplification of the target sequences, shrimp alkaline phosphatase (SAP) treatment to purify the amplicons, a single-base extension reaction, and a further resin purification step. The final products were then analyzed by matrix-assisted laser desorption/ionization time-of-flight (MALDI-TOF) mass spectrometry. Data acquisition and initial analysis were conducted with Agena Typer 4.0 Software, and any results that did not meet quality control standards were excluded from the final analysis.

### Statistical analysis

Statistical analyses were performed using IBM SPSS Statistics 26.0 and PLINK 1.9. Deviation from Hardy-Weinberg equilibrium (HWE) was assessed in the control group via chi-square testing to evaluate genotyping quality and potential population stratification. A two-sided *P*-value < 0.05 indicated statistical significance. SNP-associated risk was quantified by adjusted odds ratios (AORs) with 95 % confidence intervals (CI) through logistic regression under four genetic models: homozygous, heterozygous, dominant, and addictive. Additionally, MDR analysis (v3.0.2) was employed to investigate epistatic interactions among SNPs and their collective impact on ALL susceptibility.

## Results

### Characteristics of the study population

This population-based case-control study included 758 individuals (360 ALL patients and 398 controls). The two groups were matched for sex and ages (sex, *P* = 0.72; patients group mean age: 5.33 ± 3.38 years, control group mean age: 5.11 ± 2.31 years, *P* = 0.29). The results of HWE testing indicated that the genotype distributions of all nine genetic variants conformed to the HWE (*P* > 0.05; Table 1).

**Table 1:** Information of enrolled genetic variations.

Gene	SNP	1000 G MAF	Chromosome	Allele	*P*-value of HWE in control
*IKZF1*	rs6964823	0.37	7: 50392398	A/G	0.15
*IKZF1*	rs11978267	0.23	7: 50398606	G/A	1.00
*IKZF1*	rs11980379	0.22	7: 50402283	C/T	0.71
*IKZF1*	rs4132601	0.22	7: 50402906	G/T	0.71
*IKZF1*	rs10272724	0.21	7: 50409515	C/T	0.71
*ARID5B*	rs10994982	0.44	10: 61950345	G/A	0.32
*ARID5B*	rs10821938	0.51	10: 61965014	C/A	0.37
*CEBPE*	rs4982731	0.33	14: 23116124	C/T	0.56
*CEBPE*	rs2144827	0.21	14: 23118022	G/A	0.92

1000 G MAF, 1000 Genomes minor allele frequency; HWE, Hardy-Weinberg equilibrium.

### Association between genetic variations and risk of ALL

Logistic regression analysis revealed that four SNPs tested (rs11980379, rs4132601, rs10272724, and rs4982731) were associated with an increased risk of ALL (rs11980379 CC *vs.* TT: AOR = 2.86, 95 % CI: 1.30–6.31; rs4132601 GG *vs.* TT: AOR = 2.73, 95 % CI: 1.23–6.04; rs10272724 CC *vs.* TT: AOR = 3.92, 95 % CI: 1.83–8.43; additive model: AOR = 1.37, 95 % CI: 1.07–1.76; rs4982731 CC *vs.* TT: AOR = 2.60, 95 % CI: 1.20–5.64; TC/CC *vs.* TT: AOR = 1.45, 95 % CI: 1.06–1.98; additive model: AOR = 1.45, 95 % CI: 1.12–1.88). whereas, three SNPs (rs10994982, rs10821938 and rs2144827) were associated with a reduced risk of ALL (rs10994982 GG *vs.* AA: AOR = 0.65, 95 % CI: 0.44–0.96; AG/GG *vs.* AA: AOR = 0.72, 95 % CI: 0.53–0.98; additive model: AOR = 0.80, 95 % CI: 0.65–0.97; rs10821938 CC *vs.* AA: AOR = 0.67, 95 % CI: 0.45–1.00; AC *vs.* AA: AOR = 0.57, 95 % CI: 0.41–0.80; AC/CC *vs.* AA: AOR = 0.60, 95 % CI: 0.44–0.83; additive model: AOR = 0.80, 95 % CI: 0.66–0.98; rs2144827 GA *vs.* GG: AOR = 0.73, 95 % CI: 0.54–0.99; GA/AA *vs.* GG: AOR = 0.72, 95 % CI: 0.54–0.96; additive model: AOR = 0.80, 95 % CI: 0.65–0.99), shown in Table 2.

**Table 2:** Logistic regression analysis of association between the polymorphisms and ALL risk.

Gene	Genotype	Cases, n (%)	Controls, n (%)	AOR (95 % CI)^a^	*P*-value^a^
*IKZF1* rs6964823	GG	273 (75.8)	296 (74.4)	Reference	
	GA	72 (20.0)	90 (22.6)	0.87 (0.61–1.23)	0.43
	AA	15 (4.2)	12 (3)	1.36 (0.62–2.95)	0.44
	GA/AA	87 (24.2)	102 (25.6)	0.92 (0.66–1.28)	0.62
	Additive model			0.99 (0.75–1.30)	0.92
*IKZF1* rs11978267	AA	255 (70.8)	275 (69.1)	Reference	
	AG	88 (24.4)	112 (28.1)	0.85 (0.61–1.18)	0.32
	GG	17 (4.7)	11 (2.8)	1.67 (0.77–3.63)	0.20
	AG/GG	105 (29.2)	123 (30.9)	0.92 (0.67–1.26)	0.59
	Additive model			1.01 (0.77–1.31)	0.97
*IKZF1* rs11980379	TT	248 (68.9)	278 (69.8)	Reference	
	TC	89 (24.7)	111 (27.9)	0.90 (0.65–1.25)	0.52
	CC	23 (6.4)	9 (2.3)	**2.86 (1.30–6.31)**	**0.009**
	TC/CC	112 (31.1)	120 (30.2)	1.04 (0.77–1.42)	0.79
	Additive model			1.18 (0.91–1.52)	0.22
*IKZF1* rs4132601	TT	249 (69.2)	278 (69.8)	Reference	
	TG	89 (24.7)	111 (27.9)	0.90 (0.65–1.24)	0.51
	GG	22 (6.1)	9 (2.3)	**2.73 (1.23–6.04)**	**0.01**
	TG/GG	111 (30.8)	120 (30.2)	1.03 (0.76–1.40)	0.85
	Additive model			1.16 (0.89–1.50)	0.27
*IKZF1* rs10272724	TT	237 (65.8)	279 (70.1)	Reference	
	TC	93 (25.8)	110 (27.6)	1.00 (0.72–1.38)	0.98
	CC	30 (8.3)	9 (2.3)	**3.92 (1.83–8.43)**	**0.0005**
	TC/CC	123 (34.2)	119 (29.9)	1.22 (0.90–1.66)	0.20
	Additive model			**1.37 (1.07–1.76)**	**0.01**
*ARID5B* rs10994982	AA	126 (35.0)	111 (27.9)	Reference	
	AG	162 (45.0)	189 (47.5)	0.76 (0.54–1.05)	0.1
	GG	72 (20.0)	98 (24.6)	**0.65 (0.44–0.96)**	**0.03**
	AG/GG	234 (65.0)	287 (72.1)	**0.72 (0.53–0.98)**	**0.03**
	Additive model			**0.80 (0.65–0.97)**	**0.03**
*ARID5B* rs10821938	AA	122 (33.9)	94 (23.6)	Reference	
	AC	155 (43.1)	209 (52.5)	**0.57 (0.41–0.80)**	**0.001**
	CC	83 (23.1)	95 (23.9)	**0.67 (0.45–1.00)**	**0.05**
	AC/CC	238 (66.1)	304 (76.4)	**0.60 (0.44–0.83)**	**0.002**
	Additive model			**0.80 (0.66–0.98)**	**0.03**
*CEBPE* rs4982731	TT	234 (65.0)	290 (72.9)	Reference	
	TC	105 (29.2)	98 (24.6)	1.33 (0.96–1.84)	0.09
	CC	21 (5.8)	10 (2.5)	**2.60 (1.20–5.64)**	**0.02**
	TC/CC	126 (35.0)	108 (27.1)	**1.45 (1.06–1.98)**	**0.02**
	Additive model			**1.45 (1.12–1.88)**	**0.006**
*CEBPE* rs2144827	GG	168 (46.7)	154 (38.7)	Reference	
	GA	148 (41.1)	186 (46.7)	**0.73 (0.54–0.99)**	**0.04**
	AA	44 (12.2)	58 (14.6)	0.70 (0.44–1.09)	0.11
	GA/AA	192 (53.3)	244 (61.3)	**0.72 (0.54–0.96)**	**0.03**
	Additive model			**0.80 (0.65–0.99)**	**0.04**

AOR, adjusted odds ratio; CI, confidence interval; ALL, Acute lymphoblastic leukemia.

a Adjusted by age and sex.

To further evaluate the effect of age and sex on the risk of ALL, a stratified analysis was conducted (Table 3). Among children aged ≥ 5 years, rs11980379, rs4132601, rs10272724 increased the risk of ALL in the homozygous model (rs11980379 CC *vs.* TT: AOR = 3.65, 95 % CI: 1.28–10.43; rs4132601 GG *vs.* TT: AOR = 3.36, 95 % CI: 1.17–9.70; rs10272724 CC *vs.* TT: AOR = 3.94, 95 % CI: 1.24–12.53), and rs10821938 CA genotype was shown to reduce the risk of ALL in both the heterozygous and dominant models (CA *vs.* AA: AOR = 0.52, 95 % CI: 0.32–0.83; CA/CC *vs.* AA: AOR = 0.58, 95 % CI: 0.37–0.91). Among children aged < 5 years, the rs10272724 CC genotype (CC *vs.* TT: AOR = 3.75, 95 % CI: 1.35–10.43) and rs4982731 C allele (CT *vs.* TT: AOR = 1.77, 95 % CI: 1.10–2.85; CT/CC *vs.* TT: AOR = 1.86, 95 % CI: 1.19–2.92; additive model: AOR = 1.67, 95 % CI: 1.15–2.43) were risk factors for ALL. The rs10821938 CT/CC genotypes demonstrated a protective effect against ALL risk compared to the TT genotype (AOR = 0.62, 95 % CI: 0.39–0.99). For rs10994982, a marginally significant association with reduced ALL susceptibility was observed across multiple genetic models (GG *vs.* AA: AOR = 0.57, 95 % CI: 0.32–1.01; GA/GG *vs.* AA: AOR = 0.65, 95 % CI: 0.41–1.01; and additive model: AOR = 0.74, 95 % CI: 0.56–0.99).

**Table 3:** Logistic regression analysis of association between the polymorphisms and ALL risk by age and sex.

Gene	Genotype	Age < 5		Age ≥ 5		Males		Females	
		Ca/Co	AOR (95 % CI)^a^	Ca/Co	AOR (95 % CI)^a^	Ca/Co	AOR (95 % CI)^b^	Ca/Co	AOR (95 % CI)^b^
*IKZF1* rs6964823	GG	144/136	Reference	129/160	Reference	153/171	Reference	120/125	Reference
	AG	34/41	0.78 (0.47–1.31)	38/49	0.96 (0.59–1.56)	45/55	1.09 (0.58–1.43)	27/35	0.80 (0.46–1.41)
	AA	7/5	1.32 (0.41–4.27)	8/7	1.42 (0.50–4.01)	9/8	1.26 (0.47–3.34)	6/4	1.56 (0.43–5.67)
	AG/AA	41/46	0.84 (0.52–1.37)	46/56	1.01 (0.64–1.59)	54/63	0.95 (0.62–1.45)	33/39	0.88 (0.52–1.49)
	Additive model		0.93 (0.62–1.38)		1.05 (0.72–1.53)		0.99 (0.70–1.41)		0.97 (0.63–1.50)
*IKZF1* rs11978267	AA	133/121	Reference	122/154	Reference	147/165	Reference	108/110	Reference
	GA	45/55	0.74 (0.47–1.18)	43/57	0.95 (0.60–1.51)	46/63	0.82 (0.53–1.27)	42/49	0.87 (0.53–1.43)
	GG	7/6	1.06 (0.35–3.25)	10/5	2.52 (0.84–7.58)	14/6	2.62 (0.98–6.99)	3/5	0.61 (0.14–2.62)
	GA/GG	52/61	0.76 (0.49–1.19)	53/62	1.08 (0.70–1.67)	60/69	0.99 (0.65–1.48)	45/54	0.85 (0.53–1.36)
	Additive model		0.84 (0.58–1.23)		1.18 (0.82–1.70)		1.12 (0.80–1.67)		0.84 (0.55–1.29)
*IKZF1* rs11980379	TT	130/124	Reference	118/154	Reference	143/165	Reference	105/113	Reference
	CT	46/54	0.81 (0.51–1.29)	43/57	0.98 (0.62–1.56)	48/65	0.85 (0.55–1.32)	41/46	0.96 (0.58–1.58)
	CC	9/4	2.15 (0.64–7.15)	14/5	**3.65 (1.28–10.43)**	16/4	**4.62 (1.51–14.12)**	7/5	1.51 (0.46–4.89)
	CT/CC	55/58	0.89 (0.57–1.40)	57/62	1.20 (0.78–1.85)	64/69	1.07 (0.71–1.61)	48/51	1.01 (0.63–1.62)
	Additive model		1.01 (0.69–1.47)		1.34 (0.94–1.90)		1.26 (0.91–1.77)		1.06 (0.71–1.58)
*IKZF1* rs4132601	TT	130/124	Reference	119/154	Reference	144/165	Reference	105/113	Reference
	GT	46/54	0.81 (0.51–1.29)	43/57	0.98 (0.61–1.55)	48/66	0.83 (0.54–1.29)	41/45	0.98 (0.59–1.62)
	GG	9/4	2.15 (0.64–7.14)	13/5	**3.36 (1.17–9.70)**	15/3	**5.73 (1.63–20.19)**	7/6	1.26 (0.41–3.86)
	GT/GG	55/58	0.89 (0.57–1.40)	56/62	1.17 (0.76–1.80)	63/69	1.05 (0.70–1.58)	48/51	1.01 (0.63–1.62)
	Additive model		1.01 (0.69–1.47)		1.30 (0.91–1.85)		1.26 (0.89–1.76)		1.04 (0.70–1.54)
*IKZF1* rs10272724	TT	119/124	Reference	118/155	Reference	134/166	Reference	103/113	Reference
	CT	48/53	0.94 (0.59–1.50)	45/57	1.04 (0.66–1.64)	51/64	0.99 (0.64–1.52)	42/46	1.00 (0.61–1.65)
	CC	18/5	**3.75 (1.35–10.43)**	12/4	**3.94 (1.24–12.53)**	22/4	**6.81 (2.3–20.25)**	8/5	1.76 (0.56–5.54)
	CT/CC	66/58	1.18 (0.76–1.82)	57/61	1.22 (0.79–1.89)	73/68	1.34 (0.89–2.00)	50/51	1.08 (0.67–1.73)
	Additive model		1.34 (0.95–1.89)		1.35 (0.94–1.93)		**1.55 (1.12–2.14)**		1.13 (0.76–1.68)
*ARID5B* rs10994982	AA	67/49	Reference	59/62	Reference	82/62	Reference	44/49	Reference
	GA	83/88	0.69 (0.43–1.10)	79/101	0.82 (0.52–1.31)	88/117	**0.57 (0.37–0.87)**	74/72	1.14 (0.68–1.92)
	GG	35/45	**0.57 (0.32–1.01)**	37/53	0.73 (0.42–1.27)	37/55	**0.51 (0.30–0.87)**	35/43	0.91 (0.50–1.66)
	GA/GG	118/133	**0.65 (0.41–1.01)**	116/154	0.80 (0.52–1.23)	125/172	**0.55 (0.37–0.82)**	109/115	1.06 (0.65–1.72)
	Additive model		**0.74 (0.56–0.99)**		0.86 (0.65–1.13)		**0.69 (0.53–0.90)**		0.96 (0.71–1.30)
*ARID5B* rs10821938	AA	60/42	Reference	62/52	Reference	81/52	Reference	41/42	Reference
	CA	84/94	0.63 (0.38–1.02)	71/115	**0.52 (0.32–0.83)**	83/123	**0.43 (0.28–0.68)**	72/86	0.86 (0.50–1.46)
	CC	41/46	0.62 (0.35–1.11)	42/49	0.72 (0.41–1.25)	43/59	**0.47 (0.28–0.79)**	40/36	1.14 (0.61–2.12)
	CA/CC	125/140	**0.62 (0.39–0.99)**	113/164	**0.58 (0.37–0.91)**	126/182	**0.44 (0.29–0.67)**	112/122	0.94 (0.57–1.56)
	Additive model		0.78 (0.59–1.04)		0.83 (0.63–1.09)		**0.66 (0.51–0.86)**		1.06 (0.78–1.45)
*CEBPE* rs4982731	TT	115/137	Reference	119/153	Reference	131/171	Reference	103/119	Reference
	CT	58/39	**1.77 (1.10–2.85)**	47/59	1.06 (0.65–1.61)	66/55	**1.57 (1.03–2.39)**	39/43	0.82 (0.63–1.74)
	CC	12/6	2.38 (0.87–6.55)	9/4	2.89 (0.87–9.62)	10/8	1.63 (0.63–4.25)	11/2	**6.35 (1.38–29.33)**
	CT/CC	70/45	**1.86 (1.19–2.92)**	56/63	1.14 (0.74–1.76)	76/63	**1.57 (1.05–2.36)**	50/45	1.29 (0.80–2.09)
	Additive model		**1.67 (1.15–2.43)**		1.23 (0.85–1.79)		**1.44 (1.03–2.02)**		1.45 (0.97–2.18)
*CEBPE* rs2144827	GG	90/74	Reference	78/80	Reference	93/89	Reference	75/65	Reference
	GA	77/83	0.76 (0.49–1.18)	71/103	0.70 (0.46–1.09)	87/114	0.73 (0.49–1.09)	61/72	0.73 (0.46–1.18)
	AA	18/25	0.59 (0.30–1.16)	26/33	0.81 (0.44–1.47)	27/31	0.83 (0.46–1.51)	17/27	0.55 (0.27–1.09)
	GA/AA	95/108	0.74 (0.49–1.12)	97/136	0.73 (0.49–1.10)	114/145	0.76 (0.52–1.11)	78/99	0.68 (0.43–1.06)
	Additive model		0.78 (0.57–1.05)		0.85 (0.64–1.13)		0.86 (0.66–1.14)		0.73 (0.53–1.01)

Ca/Co, Cases number/Controls number; AOR, adjusted odds ratio; CI, confidence interval; ALL, Acute lymphoblastic leukemia.

a Adjusted by sex;

b Adjusted by age.

Stratified by sex, the result demonstrated four SNPs (rs11980379, rs4132601, rs10272724, and rs4982731) were significant associated with increased ALL risk specifically in males (rs11980379 CC *vs.* TT: AOR = 4.62, 95 % CI: 1.51–14.12; rs4132601 GG *vs.* TT: AOR = 5.73, 95 % CI: 1.63–20.19; rs10272724 CC *vs.* TT: AOR = 6.81, 95 % CI: 2.3–20.25; rs4982731 CT *vs.* TT: AOR = 1.57, 95 % CI: 1.03–2.39; CT/CC *vs.* TT: AOR = 1.57, 95 % CI: 1.05–2.36; and additive model: AOR = 1.44, 95 % CI: 1.03–2.02). Conversely, both rs10994982 and rs10821938 exhibited protective effects across all genetic models in males (rs10994982 GA *vs.* AA: AOR = 0.57, 95 % CI: 0.37–0.87; GG *vs.* AA: AOR = 0.51, 95 % CI: 0.30–0.87; GA/GG *vs.* AA: AOR = 0.55, 95 % CI: 0.37–0.82; additive model: AOR = 0.69, 95 % CI: 0.53–0.90; rs10821938 CA *vs.* AA: AOR = 0.43, 95 % CI: 0.28–0.68; CC *vs.* AA: AOR = 0.47, 95 % CI: 0.28–0.79; CA/CC *vs.* AA: AOR = 0.44, 95 % CI: 0.29–0.67; additive model: AOR = 0.66, 95 % CI: 0.51–0.86).

### MDR analysis

The susceptibility of SNPs interactions to ALL risk was evaluated by MDR analysis. As summarized in Table 4, various SNP combination models demonstrated differences in equilibrium accuracy, cross-validation consistency (CVC), and statistical significance across both training and testing datasets. Comprehensive evaluation of model stability, effect size, and generalization capability revealed that the 2-SNP, 4-SNP, and 6-SNP interaction models all exhibited potential for ALL risk prediction. The 4-SNP model (rs10994982, rs2144827, rs10272724, rs10821938) emerged as the most robust predictor, demonstrating excellent generalization with minimal discrepancy between training (0.62) and testing (0.55) balanced accuracy, perfect CVC (10/10), and a clinically relevant odds ratio of 2.67 (95 % CI: 1.98–3.60). While the 6-SNP model showed the strongest association (OR = 4.94, 95 % CI: 3.63–6.72), its clinical implementation would require validation in larger cohorts due to the complexity of multi-locus interactions and the necessity for sufficient statistical power. The 2-SNP model (rs10272724, rs10821938) represented a practical alternative with satisfactory predictive performance (testing accuracy = 0.56) and high stability (CVC = 9/10), particularly suitable for studies with limited sample sizes. These findings collectively suggest that genetic interaction patterns can effectively stratify ALL risk.

**Table 4:** The impact of SNP-SNP interactions on risk of ALL.

Models	Bal. Acc. Training	Bal. Acc. Testing	CVC	OR (95 % CI)	*P*-value
rs10821938	0.55	0.53	10/10	1.66 (1.21–2.28)	0.0018
rs10272724, rs10821938	0.57	0.56	9/10	1.76 (1.39–2.50)	< 0.0001
rs4982731, rs10272724, rs10821938	0.59	0.53	5/10	2.18 (1.62–2.95)	< 0.0001
rs10994982, rs2144827, rs10272724, rs10821938	0.62	0.55	10/10	2.67 (1.98–3.60)	< 0.0001
rs10994982, rs2144827, rs4982731, rs10821938, rs11978267	0.66	0.50	5/10	3.51 (2.60–4.74)	< 0.0001
rs10994982, rs2144827, rs4982731, rs6964823, rs10821938, rs11978267	0.70	0.55	10/10	4.94 (3.63–6.72)	< 0.0001

SNP, single nucleotide polymorphism; ALL, Acute lymphoblastic leukemia; Bal. Acc., Balanced accuracy; CVC, Cross-validation consistency; OR, odds ratio; CI, confidence interval.

**Fig. 1:**
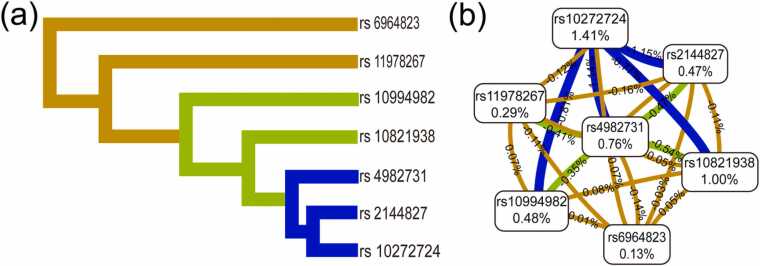
Interaction map among single-nucleotide polymorphisms (SNPs) of genes on the risk of Acute lymphoblastic leukemia (ALL). Hierarchical clustering dendrogram (a) and Kamada-Kawai (b).

Fig. 1 visually depicts the complex relationships and interaction network among susceptibility loci investigated in this study. The hierarchical clustering dendrogram (Fig. 1a) reveals distinct functional groupings among SNP sites: branch colors (blue, green, and brown) correspond to clustered subgroups. The dendrogram shows rs4982731, rs2144827, and rs10272724 (blue branch) forming a primary cluster, which then joins rs10994982 and rs10821938 (green branch); this suggests these genetically proximate variants may share functional similarities or common biological pathways in influencing ALL susceptibility. In contrast, rs11978267 and rs6964823 (brown branch) form a distinct cluster with relatively unique risk characteristics compared to other SNPs. Complementarily, the Kamada-Kawai diagram (Fig. 1b) visualizes the epistatic interaction patterns of nine genotypic SNPs in the *IKZF1*, *ARID5B*, and *CEBPE* genes through its force-directed network visualization technique: line thickness represents interaction strength, while percentages within nodes indicate each site’s respective contribution. Notably, rs10272724 (1.41 %) exhibits strong interactions (thick blue lines) with multiple loci (e.g., rs4982731, rs2144827, rs10994982, rs10821938). These patterns reflect complex epistatic interactions, where multiple genetic variants jointly determine disease susceptibility in a non-additive manner.

## Discussion

This population-based case-control association study demonstrated that seven SNPs (*IKZF1* rs11980379, rs4132601, rs10272724; *ARID5B* rs10994982, rs10821938; *CEBPE* rs4982731, rs2144827) were significantly associated with ALL susceptibility in Chinese children, and that the interaction of these SNPs may enhance the risk of ALL.

*IKZF1* is located on chromosome 7p12.2 and encodes IKAROS, a critical member of the zinc finger transcription factor family involved in hematopoietic regulation. Structurally, normal IKAROS contains four N-terminal zinc fingers (ZnF1–4) responsible for sequence-specific DNA binding at (GGGA) motifs, and two C-terminal zinc fingers (ZnF5–6) mediating homo-/hetero-dimerization with other IKAROS family proteins like HELIOS and AIOLOS [32]. This dual-domain architecture enables IKAROS to function as a master regulator of chromatin remodeling complexes, particularly through interactions with NuRD and SWI/SNF complexes, thereby modulating transcriptional programs in lymphocyte development [33], [34].

Accumulating evidence suggests that IKAROS plays a crucial role in the formation and differentiation of lymphoid lineages. Georgopoulos et al. demonstrated that IKAROS is essential for the development of all lymphoid lineages, and that lymphoid lineage cells are deficient in the presence of mutations or loss of function in the *IKZF1* gene [35]. Subsequently, gene editing and genome-wide analyses have elucidated the specific functions of the different zinc finger structural domains of IKAROS and their mode of action in lymphocyte differentiation and leukemogenesis [36], [37]. Recent data have shown that IKAROS binds to enhancer regions of key B-cell commitment genes (e.g., *EBF1*, *PAX5*) and T-cell specification factors (e.g., *GATA3*, *NOTCH1*), establishing a transcriptional network that is critical for lymphocyte fate determination [38], [39], [40]. Emerging genetic epidemiologic studies have identified *IKZF1* as a susceptibility locus for ALL [41], [42]. The five SNP loci of *IKZF1* (rs6964823, rs11978267, rs11980379, rs4132601, rs10272724) have been established as ALL risk factors in Hispanic/Latino and European populations [8], [43], [44], [45], [46]. In Chinese children, our study confirmed significant associations of rs11980379, rs4132601, and rs10272724 with ALL risk.

*ARID5B* (alternative names: *MRF2*, *DESRT*), which maps to chromosome 10q21.2, encodes a protein belonging to the ARID family. This family functions as epigenetic regulators through binding to AT-rich DNA sequences and modulating chromatin structure via interactions with partner proteins such as PHF2 to form histone demethylase complexes [47], [48]. ARID family proteins, including ARID5B protein, are critical transcriptional regulators in cell growth and differentiation, particularly in hematopoietic lineages [49]. Dysfunctions of *ARID5B* may facilitate tumorigenesis, which has been proved by high-throughput screenings for inherited predispositions or tumor genomic mutations [20]. Notably, Multiple SNPs in *ARID5B* have been reported as susceptibility markers for ALL in different regions [8], [44], [50], [51], [52], [53], and the study by Xu et al. was conducted in multiple ethnicities and explored associations in different ethnicities [43]. This study suggested that rs10994982, rs10821938 were associated with a reduced risk of ALL in the Chinese population. The potential mechanisms of *ARID5B* in leukemogenesis involve its risk variants, which may alter cis-regulatory elements [23] or disrupt transcriptional networks in hematopoietic stem cells and early lymphoid progenitors [48], [54], [55]. Functional studies in murine models demonstrate that ARID5B overexpression perturbs B-cell development, leading to reduced circulating and bone marrow B-cell counts and impaired mitochondrial oxidative metabolism in B cells [56]. Ge et al. have shown that aberrant *ARID5B* expression is linked to IKAROS dysfunction and involved in the oncogenic effect of ALL [57].

The CEBP family comprises transcription factors characterized by a highly conserved C-terminal bZIP domain that facilitates both dimerization through leucine zipper motifs and DNA binding via an adjacent basic region. Among these, *CEBPE* (located at chromosome 14q11.2) plays a pivotal role in myeloid cell differentiation, particularly in neutrophil and macrophage maturation [58]. Molecular studies demonstrate that CEBPE protein orchestrates granulopoiesis by regulating the spatial organization of specific and gelatinase granules, transcriptional activation of granule protein genes (e.g., lactoferrin, gelatinase), and delayed expression of azurophilic granule components during granulocyte development [25], [59], [60], [61]. The relationship of the *CEBPE* SNP to granulocyte diseases was extensively investigated [25], [28], [62], and its association with ALL was also conducted research consequently, which focused on rs2239633. Meta-analysis confirmed its protective effect against ALL in Caucasian and Hispanic populations [24], [29], [31]. James B. Studd and colleagues [63] employed genetic interaction analysis and molecular biology experiments to demonstrate that the rs2239630 SNP represents a functional variant contributing to genetic susceptibility to ALL. Their findings indicated that the risk allele rs2239630-A augments promoter activity and upregulates CEBPE expression, thereby promoting leukemogenesis. Our research expands this paradigm by characterizing two additional *CEBPE* loci: rs4982731-C confers significant ALL risk, while rs2144827-A exhibits protective effects, suggesting complex haplotype-specific regulation of CEBPE’s leukemogenic potential.

This study employed a systems biology approach to elucidate the polygenic architecture of ALL susceptibility through integrated SNP-SNP interaction analysis. Hierarchical clustering of risk loci revealed functionally coherent modules, where SNPs within the same branch likely share similar functions or exhibit synergistic roles. The Kamada-Kawai algorithm further validated cross-modular synergy, demonstrating that rs10272724 and rs10821938 functioned as core hubs to integrate different functional modules through multilateral linkages. Notably, these hubs mediated direct interactions between *ARID5B* (rs10994982) and *CEBPE* (rs4982731), a cross-pathway synergy that may explain the risk amplification effect observed in the multi-SNP combination model-a finding consistent with the MDR results. However, the modest interaction strength (< 1.5 %) indicates that the current model requires further validation of the biological basis for these interactions, particularly the molecular mechanisms of core node genes in transcriptional regulation, through expanded sample sizes and functional experiments.

This study has some limitations. First, the sample was recruited from two hospitals in Jiangsu Province, which may introduce regional representativeness constraints. Replication studies in geographically diverse populations would help verify the external validity of these findings. Second, the current sample size is relatively modest for robust validation of the multi-SNP models identified via MDR analysis. Larger-scale independent cohorts are needed to further evaluate these models, which would support their potential translation into ALL risk prediction frameworks. Third, the discussion on the potential biological synergies among the SNPs is based on existing studies. Future work integrating functional genomics assays (e.g., reporter gene assays or protein interaction studies) would help characterize the biological mechanisms underlying the synergistic effects of these genetic variants.

## Conclusions

This case-control study confirms the associations between specific SNPs in *IKZF1*, *ARID5B*, and *CEBPE* with susceptibility to ALL, and further highlights the amplifying effect of gene-gene interactions on disease risk. Further functional validation and large-scale prospective cohort studies are warranted to confirm these interactions and assess their potential clinical utility for risk stratification.

## Declaration of Competing Interest

The authors declare no conflicts of interest.

## Data Availability

The data that support the findings of this study are available from the corresponding author upon reasonable request.
